# Defect Studies in Thin-Film SiO_2_ of a Metal-Oxide-Silicon Capacitor Using Drift-Assisted Positron Annihilation Lifetime Spectroscopy

**DOI:** 10.3390/nano15151142

**Published:** 2025-07-23

**Authors:** Ricardo Helm, Werner Egger, Catherine Corbel, Peter Sperr, Maik Butterling, Andreas Wagner, Maciej Oskar Liedke, Johannes Mitteneder, Michael Mayerhofer, Kangho Lee, Georg S. Duesberg, Günther Dollinger, Marcel Dickmann

**Affiliations:** 1 Institute for Applied Physics and Measurement Technology, University of the Bundeswehr Munich, 85579 Munich, Germany; werner.egger@unibw.de (W.E.); peter.sperr@unibw.de (P.S.); johannes.mitteneder@unibw.de (J.M.); michael.mayerhofer@unibw.de (M.M.); guenther.dollinger@unibw.de (G.D.); marcel.dickmann@unibw.de (M.D.); 2 LSI, CEA/DRF/IRAMIS, CNRS, Ecole Polytechnique, Institut Polytechnique de Paris, 91120 Palaiseau, France; catherine.corbel@polytechnique.edu; 3 Institute of Radiation Physics, Helmholtz Center Dresden-Rossendorf, 01328 Dresden, Germany; m.butterling-2@tudelft.nl (M.B.); a.wagner@hzdr.de (A.W.); m.liedke@hzdr.de (M.O.L.); 4 Institute of Physics, University of the Bundeswehr Munich, 85579 Munich, Germany; kay.lee@unibw.de (K.L.); duesberg@unibw.de (G.S.D.)

**Keywords:** drift-enhanced positron annihilation lifetime spectroscopy, thin-films, interface defects, positronium formation, positron drift

## Abstract

This work investigates the impact of an internal electric field on the annihilation characteristics of positrons implanted in a 180(10)nm SiO_2_ layer of a Metal-Oxide-Silicon (MOS) capacitor, using Positron Annihilation Lifetime Spectroscopy (PALS). By varying the gate voltage, electric fields up to 1.72MV/cm were applied. The measurements reveal a field-dependent suppression of positronium (Ps) formation by up to 64%, leading to an enhancement of free positron annihilation. The increase in free positrons suggests that vacancy clusters are the dominant defect type in the oxide layer. Additionally, drift towards the SiO_2_/Si interface reveals not only larger void-like defects but also a distinct population of smaller traps that are less prominent when drifting to the Al/SiO_2_ interface. In total, by combining positron drift with PALS, more detailed insights into the nature and spatial distribution of defects within the SiO_2_ network and in particular near the SiO_2_/Si interface are obtained.

## 1. Introduction

Field-effect transistors are the building block of modern electronics. More recently, thin-film transistors (TFT) have gained in relevance. Such devices have several advantages over conventional Metal-Oxide-Semiconductor transistors (MOSFET) [1]. These devices can be constructed from a variety of materials [2] and are utilized in the fabrication of sensors, detectors, integrated circuits, and displays [3]. One of the limiting factors of their device performance and reliability is the dielectric-semiconductor interface. At the interface, structural defects lead to interface states, which decrease carrier mobility and act as carrier traps [1,2]. With increasing performance demands and the continuous development of semiconductor device concepts, such as 3D integrated transistors [4] and 2D devices [5], the choice of dielectric materials has become increasingly important. Each new oxide system—such as HfO_2_ [6], or Gd_2_O_5_ [7]—introduces its intrinsic defect configuration as well as unique interface-related defect structures. To optimize and better understand the fabrication processes of these oxide layers, it is important to know the origin of defects in order to control their formation for specific applications. In the following, a method is presented that has the potential to characterize defects in ultra-thin oxide layers, and most importantly, at their interfaces.

Positron Annihilation Lifetime Spectroscopy (PALS) is a unique, non-destructive analytical technique used to characterize defects in various fields of materials science [8,9,10]. Analyzing the positron lifetime in a material allows for the identification of defect size and type, which are characteristic of the specific material system. For thin films or layered material systems, PALS based on slow-positron beams is an indispensable tool for depth-resolved defect characterization [11]. However, due to the width of the slow positron implantation profile, the detection of buried ultra-thin films of thicknesses of only a few nanometers and even interfaces can be difficult. It is possible to overcome this limitation by combining PALS with slow positrons in the keV range and a simultaneous application of a sample bias, which can lead to strong electric fields inside the material. An electric field influences the positron diffusion by adding a drift velocity ${\vec{v}}_{d}=\mu_{+}\vec{E}$, with $\mu_{+}$ being the positron mobility in the material. The drift can thus be exploited to transport positrons to the desired region. The simplest geometry and one of the most researched thin-layered systems is the Metal-Oxide-Silicon (MOS) capacitor [12,13,14,15]. One of the first to demonstrate drift-assisted Doppler broadening measurement on a MOS capacitor was Uedono et al. [16] followed by other groups who used this technique to gain insight into the structure of the SiO_2_ layer and especially the SiO_2_/Si interface [17,18,19,20,21,22]. Thus, the Metal–SiO_2_–Si capacitor is well-suited for testing and validating the new positron annihilation technique.

All previous drift-assisted PAS measurements are based on Doppler broadening spectroscopy, from which it is difficult to derive quantifiable information about the defect type, size, and concentration. Positron Annihilation Lifetime Spectroscopy (PALS) offers a precise method to determine the number of exponential decay components arising from positron annihilation in various quantum states. Currently, only two pulsed low-energy positron beam instruments—PLEPS [23] and MePS [24]—offer the time resolution and peak-to-background ratio required to perform near-surface PALS measurements under bias voltage conditions with both high reproducibility and reliability. This work presents the first beam-based drift-assisted PALS experiment on a thermally grown SiO_2_ layer on a Si(100) substrate.

## 2. Materials and Methods

### 2.1. Metal-Oxide Silicon System

The SiO_2_ was thermally grown on a 500μm p-doped silicon(100) substrate with a doping concentration of $1.2\times 10^{15}\;{\mathrm{cm}}^{-3}$. The thermal oxidation process was carried out at 950°C in H_2_O atmosphere for 75min. The resulting oxide layer has a thickness of 180(10)nm. No post-oxidation annealing was performed. An aluminum gate with an area of $10\times 10\;{\mathrm{mm}}^{2}$ and a thickness of 40(5)nm was vapor-deposited onto the oxide using a physical vapor deposition (PVD) evaporation system. The back of the silicon wafer was also vapor-deposited with a thin aluminum layer, which serves as a back contact. Standard Capacitance-Voltage (CV) profiling of the Metal-Oxide-Silicon (MOS) system revealed a flat-band voltage of −0.9V and a surface state density of $10^{10}$–$10^{11}\;{\mathrm{cm}}^{-2}eV^{-1}$ at the SiO_2_/Si interface.

A bare oxide-silicon stack from the same wafer was also prepared to determine the annihilation characteristics of the oxide in the absence of the Al layer. The reference stack will be referred to as the bare oxide, and the oxide layer of the MOS capacitor will be referred to as the buried oxide.

### 2.2. Positron-Annihilation Lifetime Spectroscopy (PALS)

When pulsed low-energy positrons are implanted into a material, their depth distribution follows a Makhovian profile, which mainly depends on the material density and the incident positron energy [11]. In the case of positron implantation at 4keV into amorphous SiO_2_, this distribution extends from the surface to about 300nm into the oxide, with a mean implantation depth of 124nm. Within this range, 99% of all positrons are contained.

After implantation and thermalization in the lattice, the positron diffuses through the material until it becomes trapped at a defect site, provided that the defect concentration is higher than $1\times 10^{16}\;{\mathrm{cm}}^{-3}$ [25]. The positron eventually annihilates with an electron of the surrounding lattice. The time between implantation and annihilation is called the positron lifetime. This lifetime is inversely related to the local electron density, which varies between the bulk and defect regions. Therefore, the measured lifetime provides insight into the material’s defect structure and its defect density. A detailed discussion of positron–matter interactions is beyond the scope of this work. For a comprehensive overview of the underlying physical principles, we kindly refer the reader to the relevant literature, such as [26].

The PALS spectra were recorded as a function of implantation energy in the range of 1–11keV at the Mono-energetic Positron Source (MePS) at the Helmholtz Center Dresden-Rossendorf [24]. At least $1\times 10^{7}$ counts were accumulated per spectrum, and the peak-to-background ratio of the recorded spectra was higher than $1\times 10^{7}$:1. The total time resolution of MePS (pulsing system + detector) was 230ps (FWHM) during the experiment. The time resolution was stable over time and the range of implantation energies. A three-component decomposition was used to fit all lifetime spectra into three resolved lifetime components with a good accuracy of $\chi^{2}\leq 1.20$ for the unconstrained fits. The three-component decomposition provided the best statistical fit, while other combinations either failed to converge or resulted in higher $\chi^{2}$ values. The uncertainties reported in this work for the lifetime and intensity values correspond to the statistical errors from the fitting procedure. The starting values and the channel ranges were kept constant for all spectra.

For the drift-assisted PALS experiment, positron lifetime spectra were recorded as a function of implantation energy at varying applied voltages from −30V to 30V. This voltage range proved stable against breakdown, and the maximum voltages generate a sufficiently strong electric field of over $1\;MV{\mathrm{cm}}^{-1}$ in the oxide layer, where a strong drift effect on positrons has been demonstrated in previous Doppler experiments [19,22].

An Iseg DPS High Precision HV module was used to apply the gate bias. Table 1 lists the applied gate voltages $U_{g}$ along with the corresponding electric fields $E_{\mathrm{ox}}=U_{g}/d_{\mathrm{ox}}$ across the buried oxide, where $d_{\mathrm{ox}}$ is the oxide thickness.

**Figure 1:**
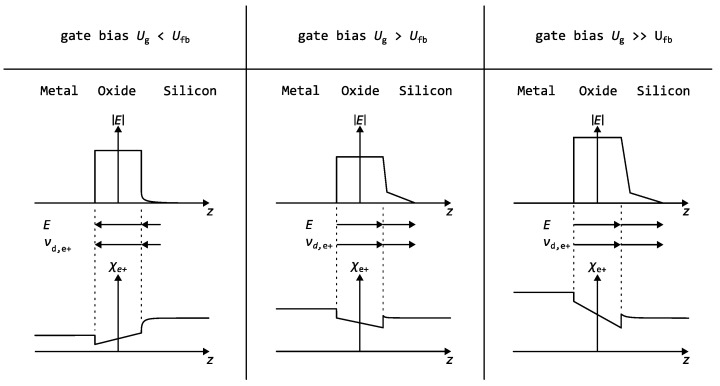
Schematic presentation of the electric field, its direction, the drift direction, and the positron affinity in the MOS layers for different gate voltages relative to the flatband voltage $U_{\mathrm{fb}}$.

The voltage $U_{g}$ was applied between the Al gate on top of the SiO_2_ layer and the ohmic Al contact at the back of the Si substrate, which was grounded. Due to the mismatch between the work functions of Al and Si, a built-in potential is established. This potential gives rise to an electric field across the oxide and causes band bending in the near-surface region of the semiconductor, even when no external voltage is applied. By applying the flatband voltage $U_{\mathrm{fb}}$, this built-in potential can be compensated, resulting in the flatband condition, which represents the electrically undisturbed, field-free state of the MOS structure. Figure 1 shows a schematic illustration of how different gate voltages $U_{g}$, relative to the flatband voltage $U_{\mathrm{fb}}$, influence the electronic behavior of the MOS structure. It shows the electric field in the MOS capacitor, including the directions of the electric field, and the resulting positron drift for the different bias conditions. The positron affinity $\chi_{+}$ of the defect-free material layers is depicted schematically at the bottom of Figure 1 [27].

## 3. Results and Discussion

### 3.1. Comparison of the Decay Spectra for the Bare and Buried Oxide

**Figure 2:**
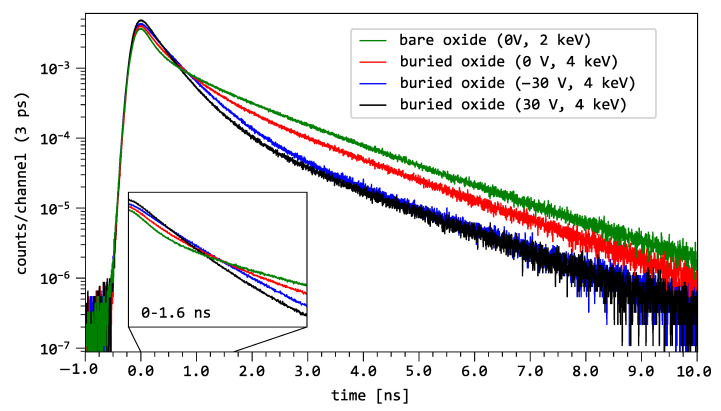
PALS spectra of the bare oxide at 2keV and the buried oxide at 4keV for the three distinct bias voltages conditions, $U_{g}=-30$, 0, 30V in the range from −1ns to 10ns. The spectra are enlarged for short decay times (1–1.6ns). All spectra were measured with the same statistics of $1\times 10^{7}$ counts/spectrum and are normalized to their area.

Figure 2 shows the comparison of the normalized positron (e^+^) lifetime spectra of the bare oxide at 2keV and the MOS structure at 4keV for three bias voltage conditions: open circuit 0V, −30V and 30V. The distinct bias conditions provide direct evidence of the impact of the electric field on the oxide’s annihilation characteristics. The comparison of the decay spectra for $U_{g}=0\;V$ for bare oxide on silicon at 2keV (green curve) and buried oxide at 4keV (red curve) shows that the decay in the time range of 5–10ns is affected by the deposition of the aluminum gate on the buried oxide. The presence of the Al gate lowers the intensity of the longest component without any significant effect on the decay slope and thus on the positron lifetime in the SiO_2_ layer.

It can be observed by comparing the red, blue and black curves, that the intensity of the longest component in the 5–10 ns range decreases with the magnitude of $U_{g}$, from 0–30 V, independent of the polarity. Remarkably, the slope remains quasi-constant, independent of the magnitude and polarity of $U_{g}$. In the 1–1.6 ns range, the curves of the MOS capacitor show a different trend depending on $U_{g}$ as depicted in the inlay of Figure 2. This indicates that for short decay times, the effect of the gate voltage is dependent on the magnitude and polarity of $U_{g}$ and leads to different annihilation characteristics. This suggests the presence of distinct positron annihilation modes near the Al/SiO_2_ and SiO_2_/Si interfaces, respectively.

### 3.2. Positron Annihilation Quantum States in the Bare Oxide Stack

#### 3.2.1. Annihilation Characteristic of the Bare Oxide Layer

**Figure 3:**
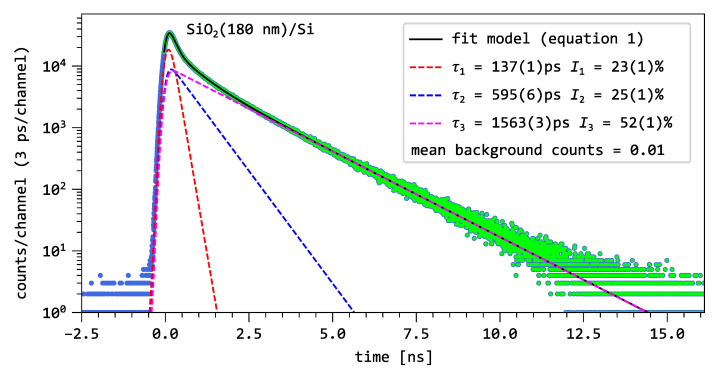
Example decay spectrum with a three-component decomposition using Equation (1) for the bare oxide layer at an implantation energy of 2keV. The blue dots represent the experimental data, the green dots indicate the data range used in the fit model, and the colored dashed lines correspond to the exponential decay components extracted from the fit.

Figure 3 shows a decay spectrum of the bare oxide layer analyzed with a pulsed positron beam of 2keV. Due to the low mobility of positrons in the oxide, their annihilation occurs predominantly within the SiO_2_ near-surface region, resulting in minimal contributions from the silicon substrate or the surface itself. This allows us to observe the undisturbed annihilation characteristics of SiO_2_. The blue dots correspond to the measured decay spectrum. The spectrum with the green dots given by a limited time window is fitted by a model function $D_{\exp}$ of the form:

(1) $$D_{\exp}\left(t\right)=\left(IRF*\sum_{i=1}^{3}\frac{I_{i}}{\tau_{i}}e^{t/\tau_{i}}\right)+B$$

In the actual spectrum, $D_{\exp}$ is decomposed into a sum of three exponential decay components of lifetimes $\tau_{i}$ and intensities $I_{i}$, convoluted with the instrument resolution function IRF. In addition, a constant background *B* is added. Accurate extraction of the lifetime components requires optimizing the time resolution to be as small as possible and determining the IRF as precisely as possible. The colored dashed lines correspond to the exponential decay components extracted from the fit.

The three lifetime components, indicated by colored dashed lines in Figure 3, include two lifetimes above 500ps identified as $\tau_{2}=595\left(6\right)\;\mathrm{ps}$ and $\tau_{3}=1563\left(3\right)\;\mathrm{ps}$, which together contribute 77% to the total spectrum. The shortest lifetime, $\tau_{1}=137\left(1\right)\;\mathrm{ps}$, accounts for roughly a quarter of the resolved annihilation signal.

Positron lifetimes above 500ps can be attributed to the annihilation of ortho-positronium (o-Ps), a hydrogen-like bound state of an electron and a positron [28]. Positronium formation is commonly observed in nanometer or sub-nanometer voids in materials such as amorphous insulators and polymers. Two spin states of Ps exist: the singlet state, known as para-Ps, which annihilates via two-gamma emission with a vacuum lifetime of 125ps, and the triplet state, known as ortho-Ps, which annihilates via three-gamma emission with a vacuum lifetime of 142ns [29]. However, in condensed matter, the lifetime of o-Ps is typically reduced to a few nanoseconds due to the processes of positronium pick-off annihilation with the surrounding electrons of the material by two-gamma emission or other quenching mechanisms [30,31,32]. Spin statistics demands the formation ratio of p-Ps to o-Ps in vacuum to be 1:3 [29,33].

It follows that the long component, $\tau_{3}=1558\left(3\right)\;\mathrm{ps}$ and $I_{3}$ = 52(1)%, is attributed to the pick-off annihilation of ortho-positronium (o-Ps) in open-volume defects. The intermediate component, $\tau_{2}=585\left(6\right)\;\mathrm{ps}$ and $I_{2}$ = 25(1)%, includes contributions from both o-Ps pick-off annihilation within the oxide layer and the annihilation of free positrons. The shortest component, $\tau_{1}=136\left(2\right)\;\mathrm{ps}$ and $I_{1}$ = 23(1)%, is related either to the self-annihilation of para-positronium (p-Ps) in the oxide, or, to the annihilation of free positrons, depending on the implantation energy. Since all lifetime components originate at least partly from positronium annihilation, it can be concluded that positronium formation is a dominant physical process in the oxide.

#### 3.2.2. Depth Profile of the Bare Oxide Sample

**Figure 4:**
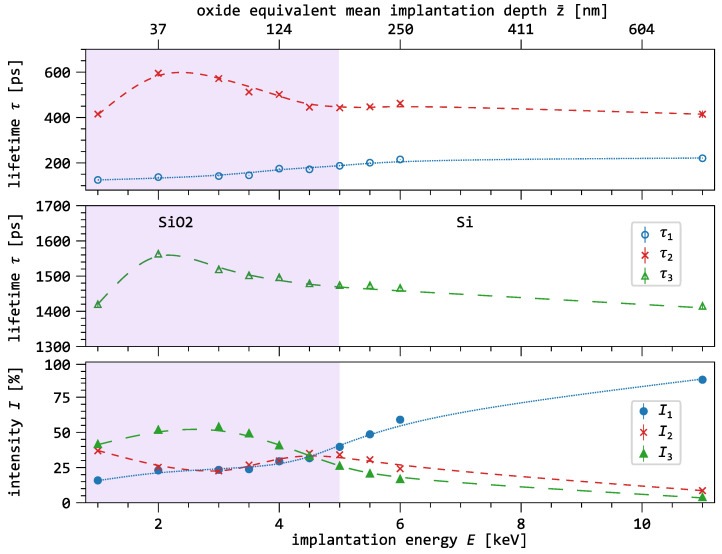
Results of the three-component analysis of the bare oxide as a function of the implantation energy. As a depth reference, the oxide-equivalent mean implantation depth $\bar{z}$ is depicted on the secondary axis.

Figure 4 shows the three-component decomposition as a function of the implantation energy. As a depth reference, the oxide-equivalent mean implantation depth $\bar{z}$ is shown on the secondary x-axis. The oxide layer is highlighted in purple, and the lines connecting the data points serve as guides to the eye.

We observe that, with increasing implantation energy, the lifetime of the first component (blue) increases from 137ps to 215ps. The largest change occurs between 4keV and 6keV. The intensity of the first component also increases steadily with increasing implantation energy, with the most pronounced rise occurring at the transition from the oxide to the semiconductor layer.

The lifetime of the second component (red) initially increases between 1keV and 2keV and then continuously decreases until the energy is reached where the mean penetration depth matches the interface of the oxide. For higher energies, it remains nearly constant. The intensity of the second component shows maxima at 1keV and 4.5keV. Its intensity decreases slightly inside the oxide, while it continuously decreases outside the oxide.

The lifetime of the third component (green) first increases in the same energy range as the second component and then steadily decreases with increasing implantation energy. Its intensity initially increases slightly and reaches a maximum of $I_{3}$ = 52% between 2keV and 3keV. As the oxide/semiconductor interface is approached, its intensity decreases to 5%.

It is well documented in the literature that positronium (Ps) annihilation is generally an annihilation mode that does not occur in conductive materials such as aluminum and silicon [30]. Thus, Ps annihilation mode as para-Ps or ortho-Ps with the relation of $I_{o}/I_{p}=3$, can be safely attributed to the SiO_2_ or SiO_2_/Si interface.

This interpretation is supported by the parallel behavior of $I_{1}$ and $I_{3}$ at implantation energies below 3keV in Figure 4. Additionally, the ratio $I_{3}/I_{1}\approx 2.5$ aligns with the expected formation ratio. With increasing positron fraction in the silicon—above 4keV—the intensity of the o-Ps component decreases and $I_{1}$ increases sharply and approaches the lifetime expected in defect-free silicon [34,35].

At an implantation energy of 1keV, surface effects are still visible in all three components. In particular, $\tau_{2}$ shows a typical surface lifetime of about 400ps [36,37]. As the energy increases, $I_{2}$ decreases because fewer positrons annihilate in surface states. However, between 3keV and 5keV, $I_{2}$ increases while simultaneously the o-Ps intensity $I_{3}$ decreases as the fraction of positrons in the substrate increases and more positrons can reach the SiO_2_/Si interface via back-diffusion. Above 5keV, $I_{2}$ drops to nearly zero as the positron fraction in the oxide and the near-surface interface region decreases with the implantation energy. The trends observed in $I_{2}$ and $I_{3}$ suggest that open-volume defects dominate the microstructure of the oxide, while no such defects are present in the silicon.

The lifetime value of $\tau_{1}$ is longer then the vacuum lifetime (125ps) of p-Ps. This indicates that other annihilation modes could contribute to the short lifetime component. They most likely arise from the 2γ annihilation mode of unbound positron-electron pairs, i.e., free positron annihilation. This gives rise to an unknown component $\tau_{1}^{\prime }$. We use a simple model, which utilizes the positron formation ratio to extract the unknown lifetime $\tau_{1}^{\prime }$ as follows:

(2) $$\tau_{1}^{\prime }=\left(I_{1}\tau_{1}-I_{p}\tau_{p}\right)/\left(I_{1}-I_{p}\right)$$

This model assumes that the p-Ps lifetime is equal to the vacuum lifetime of $\tau_{p}=125\;\mathrm{ps}$ with an intensity $I_{p}=I_{3}/3$. For the lifetime component, $\tau_{1}^{\prime }$ and $I_{1}^{\prime }$, the calculated values extracted from Equation (2) are $\tau_{1}^{\prime }=169.9\;\mathrm{ps}$ and $I_{1}^{\prime }$ = 5.7%.

Similar lifetimes and intensities were reported for crystalline, vitreous bulk [38] or thin layers [20]. The most striking comparison to the literature is for the calculated lifetime component, $\tau_{1}^{\prime }$ and $I_{1}^{\prime }$. Such a lifetime of about ≈160ps has been reported for an crystalline SiO_2_ with a much higher intensity of about 25% [38]. This leads to the assumption that in the SiO_2_ system investigated in this work, some precipitates of crystalline silica are embedded in the amorphous SiO_2_ network.

### 3.3. Positron Annihilation Quantum States in the MOS Capacitor

**Figure 5:**
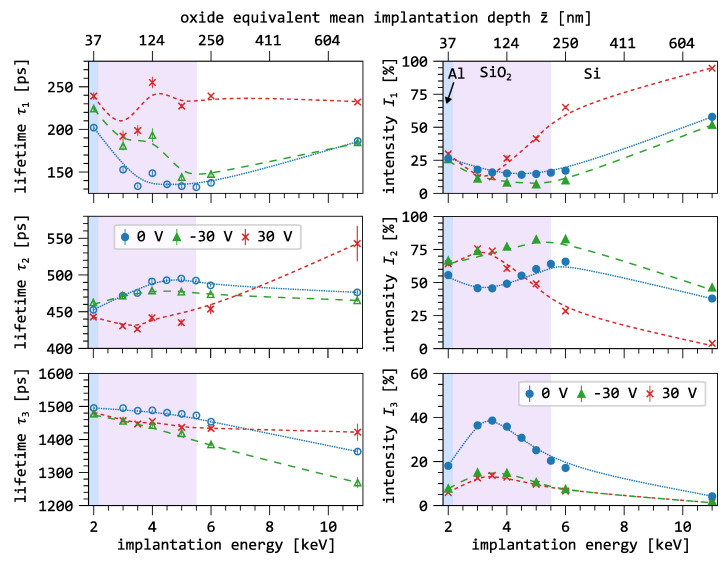
Results of the three-component analysis of the buried oxide lifetime spectra as a function of implantation energy for three different applied gate voltages. As a depth reference, the oxide equivalent mean implantation depth $\bar{z}$ is depicted on the secondary axis. The different layers are indicated by colored boxes: blue for the Al layer, violet for the SiO_2_ layer, and white for the silicon substrate. The dashed lines between the data points are guides to the eye.

The evolution of the lifetime components in the different layers of the MOS capacitor can be seen in Figure 5. We investigate the MOS capacitor under three different gate voltages, namely 0V and the two extreme cases of 30V and −30V. As a depth reference, the oxide-equivalent mean implantation depth $\bar{z}$ is shown on the secondary axis, and the different MOS layers are highlighted using colors (blue for aluminum and purple for the oxide). In this graph, the different colors of the data points correspond to the different gate voltage configurations. The lines connecting the data points are intended as eye guides.

Under open-circuit conditions, the first lifetime component decreases from 202(2)ps at 2keV to a minimum of 134(4)ps, then increases for energies above 5.5keV. Its intensity follows a parabolic trend, with a minimum of 14% at 4.5keV. The second component increases from 453(3)ps in the aluminum (2keV) to 495(2)ps in the oxide, then slightly decreases for higher energies. Its intensity reaches a maximum of 66(1)% at 6keV, followed by a decrease to 38(1)%. The third component lifetime decreases from 1495(4)ps to 1363(10)ps with increasing energy, while its intensity peaks at 39(1)% in the oxide and drops to 4(1)% at 11keV.

Under negative gate bias (−30V), the first component behaves similar to the open circuit condition but with higher lifetimes between 2keV and 6keV. We observe a parabolic intensity minimum at 5keV and for higher energies, the intensity increases to a maximum of 52(1)% at 11keV. The second component remains nearly constant between 470ps and 478ps, with intensity increasing to 83(1)% at the oxide–semiconductor interface, then decreasing to 46(1)%. The third component lifetime decreases to 1270(17)ps, while its intensity follows a similar trend as under open-circuit conditions, but with a lower peak at 15(1)%.

With positive gate bias (30V), the first component reaches a minimum of 192(7)ps at 3.5keV, then increases up to 240ps. Its intensity drops to 12(1)%, then rises to 95(1)% at the highest energy. The second component stays around 435ps below 5.5keV and increases to 543(24)ps, with intensity peaking at 75(1)% before dropping to 4(1)%. The third component lifetime decreases moderately to 1422(25)ps, and its intensity remains similar to the negative bias case, with a slightly lower maximum of 13(1)%.

Figure 5 unambiguously shows the effect of the electric field on the lifetime components as a function of implantation energy. The blue curve (0V) serves as the reference measurement and shows that the annihilation characteristics are only affected by diffusion. In the oxide, the diffusion is very limited with a diffusion length of ≈10 nm [39] and thus, oxide-thermalized positrons annihilate close to their location of thermalization. We can summarize the annihilation modes based on their energy as follows:

- free positron annihilation in the Al-gate for $E_{\mathrm{imp}}\leq 2\;\mathrm{keV}$.
- positronium and free positron annihilation in the oxide between $2\;\mathrm{keV}\leq E_{\mathrm{imp}}\leq 5\;\mathrm{keV}$.
- free positron annihilation in the substrate for $E_{\mathrm{imp}}\geq 5.5\;\mathrm{keV}$.

All lifetimes and intensities from the different gate voltage conditions show a similar starting point at 2keV. However, the variation in τ and *I* is the first indicator of a positron drift. We can conclude this since a fraction of positrons is already implanted into the oxide and thus drifts to either one of the interfaces. The lifetimes and intensities for energies between 2.5keV and 4.5keV can be attributed to positrons mainly annihilating in the oxide of the MOS capacitor. For 0V, the intensity of the o-Ps component is highest, effectively reducing the contribution of the other two annihilation modes. When a gate voltage is applied, the intensity of the o-Ps component reduces significantly by ≈60%. This decrease depends only on the electric field strength and not on the direction. We can conclude that the application of the electric field greatly inhibits the formation of o-Ps.

Another observation in Figure 5 is the deviation of $I_{1}$ and $I_{2}$ at around 3.5keV for the two biased measurement. First, they show the same trend, suggesting that positrons see the same amount of defects, but with different defect sizes. This suggests that vacancy clusters might be the main defect type in the SiO_2_ layer. The size of these vacancy clusters varies with the location of positron annihilation within the oxide. They become smaller as the positrons annihilate closer to the SiO_2_/Si interface. In Figure 5 above 3.5keV, the intensities, $I_{1}$ and $I_{2}$, of the 30V measurement starts to differ from the −30V measurement. With increasing implantation energy, a larger fraction of positrons is implanted into the silicon substrate. This fraction then drifts in the near-surface electric field of the silicon layer. For negative gate voltages, the positrons in the oxide still drift to the gate electrode, and positrons in the substrate get drifted back to the SiO_2_/Si interface, increasing the intensity $I_{2}$ of vacancy clusters present at the interface. For positive gate voltages, positrons in the oxide get drifted to the SiO_2_ interface, and substrate-implanted positrons get drifted into the silicon bulk. However, the increase in positron fraction in the substrate alone does not fully explain the intensity change between 3.5keV and 4keV:

The positron fraction, $F_{ox}$, that stops in the oxide remains constant at approximately 80% for both energies. The positron fraction, $F_{Si}$, stopping in the silicon substrate increases by 6% from 3.5keV to 4keV. This increase is smaller than the observed change in intensity, $\Delta I_{1}\approx$ 14(1)%, from 12(1)% to 26(1)%. Therefore, the rise in intensity cannot be attributed solely to annihilation in the silicon substrate. Instead, this change likely includes contributions from annihilation within the oxide. Given its dependence on both implantation depth and electric field strength, this annihilation mode is likely associated with the microstructure near the SiO_2_/Si interface.

Above implantation energies of 4.5keV, the intensity of the first component begins to align with the increasing positron fraction in the substrate, indicating that both lifetimes and intensities are primarily influenced by the electric field in the near-surface region of the silicon layer. At 0V and −30V, positrons reach the interface through back-diffusion and drift, respectively. At 30V, Figure 5 clearly shows a trend consistent with defect-free bulk silicon, as positrons are drifted deeper into the substrate. The drift behavior in the silicon substrate will be discussed in a separate publication.

### 3.4. Positron Annihilation Quantum States in the Buried Oxide of the MOS Capacitor

The energy-dependent data in Figure 5 suggest a change in annihilation characteristics, possibly linked to the SiO_2_/Si interface. To investigate the field-induced variations in the relative contributions of Ps and free e^+^ annihilation, we need to examine how the three lifetime components depend on the applied gate voltage $U_{g}$.

**Figure 6:**
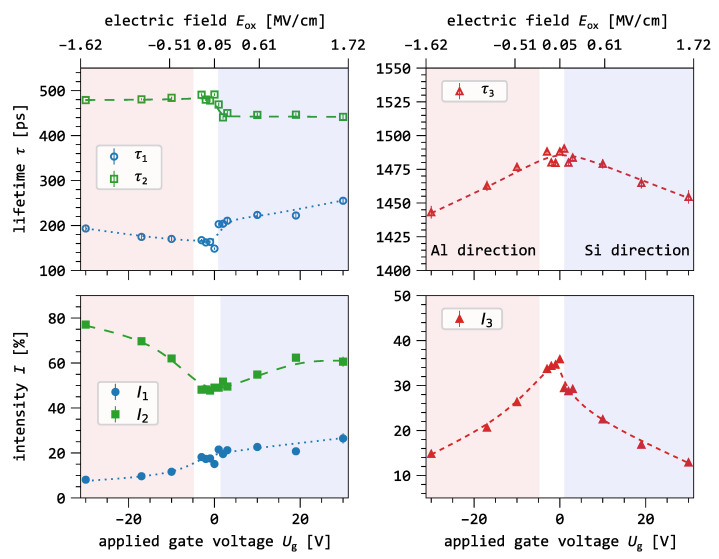
Results of the three-component analysis of the lifetime spectra as a function of the applied gate bias at 4 keV. The drift directions are divided into Al direction (red), flatband range (white) and Si direction (blue). The theoretical electric field across the oxide is shown on the secondary axis. The experimental data are also presented in Table 2.

Figure 6 presents the results of the lifetime analysis as a function of gate voltage for an implantation energy of 4keV. At that energy the mean implantation range is in the middle of the SiO_2_ layer, and about ≈80% of the positrons are stopped in the oxide layer. Thus, the number of positrons affected by the drift is maximized. The secondary axis indicates the corresponding electric field within the SiO_2_ layer derived from the applied gate voltage. The different drift regimes (see Figure 1) are highlighted using colored boxes: red (Al direction) drift to Al/SiO_2_ interface; white (flatband range) no distinct drift direction; blue (Si direction) drift to SiO_2_/Si interface.

The shortest lifetime component (blue dots), $\tau_{1}$, exhibits a distinct minimum at $U_{g}=0\;V$ of $\tau_{1}=149\left(5\right)\;\mathrm{ps}$. As the drift toward the Al gate increases (from $U_{g}=-30\;V$ to 0V), $\tau_{1}$ increases to 195(8)ps. In the opposite drift direction—toward the Si substrate –, $\tau_{1}$ increases sharply to 203(4)ps at $U_{g}=1\;V$, and continues rising to 255(7)ps at $U_{g}=30\;V$. The intensity $I_{1}$ increases gradually from 10(2)% at $U_{g}=-30\;V$ to a maximum of 23(2)% at $U_{g}=30\;V$.

The intermediate lifetime (green squares), $\tau_{2}$, remains relatively stable in both drift directions. It averages 483(9)ps for negative voltages and unter flatband condition and drops to 445(10)ps for positive voltages. However, its intensity, $I_{2}$, decreases significantly from 77% to 48% as $U_{g}$ increases from −30V to −3V. For gate voltages in the flatband range, $I_{2}$ stabilizes at approximately 49%. As the field increases in the Si direction, $I_{2}$ rises again and levels off at around 60% between $U_{g}=20\;V$ and 30V.

The longest lifetime component (red triangles), $\tau_{3}$ peaks at 1490(5)ps within the flatband range. Outside this range, in both the Al direction and Si direction, $\tau_{3}$ decreases linearly by about 3%. The corresponding intensity, $I_{3}$, also drops symmetrically with increasing field strength. It declines from 36% at $U_{g}=0\;V$ to 15% at $U_{g}=-30\;V$ and to 13% at $U_{g}=30\;V$.

#### 3.4.1. Drift Transport in SiO_2_

Two key considerations govern the drift in the oxide layer of the MOS capacitor. First, the electric field can only act on free positrons. Second, the difference in positron affinities between the oxide and the adjacent layers—illustrated in Figure 1—creates a potential well that confines positrons within the oxide [27,40,41]. Thus, the kinetic energy gained from the electric field used in this experiment is insufficient to overcome this drift barrier with ΔE≈4eV. We use transport estimations similar to charge carriers in semiconductors or metals and calculate the transit time $t_{d}$ a free positron needs to traverse the 180(10)nm oxide layer in an electric field of $1.6\;\mathrm{MV}{\mathrm{cm}}^{-1}$. The most recent calculation of the positron mobility in SiO_2_ was reported to be $\mu_{+}=1.20\left(9\right)\;{\mathrm{cm}}^{2}V^{-1}s^{-1}$ by Petkov et al. [22]. This mobility value results from fitting the drift-diffusion equation for positrons to the experimental Doppler broadening spectroscopy data. The authors also note that the determination of the diffusion constant through this fitting procedure is subject to significant uncertainty, which in turn leads to a large uncertainty in the extracted mobility. This, along with reports of much lower mobility values in the literature [17], suggests that either the fitting method or the oxide fabrication process has a substantial impact on the positron mobility. Therefore, these drift estimates should be regarded as qualitative and interpreted with caution.

With an electric field of $E=1.6\;\mathrm{MV}{\mathrm{cm}}^{-1}$, we can calculate the mean drift velocity of positrons $\langle v_{d}\rangle =\mu_{+}E=1.92\times 10^{4}\;{\mathrm{ms}}^{-1}$. Such a velocity results in an oxide transit time of $t_{d}\approx 9\;\mathrm{ps}$. This would suggest that positrons are transported rapidly through the oxide. As Petkov et al. already discussed, the electric field results in a minor addition to the total kinetic energy of thermalized positrons (≈5% in our case) and thus the de Broglie wavelength is still larger than the dimension of the defects and trapping should not be influenced drastically by the drift. However, the experimental results suggest otherwise, since a significant change in the annihilation characteristics is observed even at moderate fields (see Figure 6). This was explained by elastic scattering dominating over inelastic scattering processes, i.e., the excitation of transversal optical phonons [22]. As a result, each scattering event results in insufficient energy loss, requiring multiple interactions before a positron can be effectively trapped at a defect site. Moreover, the electric field within the oxide continuously accelerates positrons between scattering events, further reducing the likelihood of trapping. For weak electric fields, the flatband range in Figure 6, the drift effect is explained by the transport of epithermal positrons, effectively shifting the implantation profile before the thermalization is completed [22].

#### 3.4.2. Drift Effect on Positronium Annihilation

First, we give a comparison between the o-Ps annihilation in the bare oxide and the buried oxide at an applied gate voltage of 0V. The contribution of the ortho-positronium mode is maximal when the fraction of oxide positrons is maximal and the internal electric field is small or zero. The small variation of the lifetime (≈5%) between the buried oxide and the bare oxide at 0V indicates that the o-Ps pick-off process is similar for both oxides. The huge relative difference in intensity (≈16%) suggests that the o-Ps annihilation is suppressed in the buried oxide. An explanation for the observed reduction in o-Ps intensity is the superposition of two contributing factors: First, the existing intrinsic electric field at 0V in the buried oxide. Second, the fraction of positrons not thermalized in the oxide.

The application of a gate voltage strongly reduces ortho-positronium (o-Ps) annihilation, regardless of whether the voltage is positive or negative. This is shown by the decrease in o-Ps intensity, $I_{3}$, in Figure 6 as the electric field becomes stronger. The relative reduction is 64% from $I_{3}$ = 36(1)% to 13(1)%. This reduction can be explained by charge separation, where the electric field separates the free electrons—generated during the thermalization of the positron—from the positron itself before positronium formation can occur [42]. In comparison, the change in o-Ps lifetime with applied voltage is small, only about 2%. A proportional reduction is expected for the intensity of the para-positronium (p-Ps) component. Figure 6 shows this is true for negative, but not for positive voltages. For positive voltages, the increase in $I_{1}$ suggests that another positron annihilation process becomes stronger as positrons drift toward the SiO_2_/Si interface. However, the lifetime $\tau_{1}$ increases for both polarities to a lifetime higher than 125ps. This increase in $\tau_{1}$ indicates that a new annihilation mode becomes observable, which was hidden by Ps formation at zero-field conditions and now dominates when Ps formation is suppressed. The asymmetric change in $I_{1}$ suggests that this annihilation process occurs more often near the SiO_2_/Si interface.

The continuous reduction of the o-Ps annihilation is observed as the internal field of the SiO_2_ layer increases to field strengths above ≈0.2MV/cm (see Table 2). This is in agreement with previous investigations in α-SiO_2_ and polymer materials [42,43]. The Ps suppression is consistent with an internal field that either inhibits Ps formation during thermalization or during the trapping process. However, the lifetime spectra alone do not provide sufficient information to unambiguously identify the dominant process.

#### 3.4.3. Free Positron Annihilation Induced by e^+^ Drift

**Figure 7:**
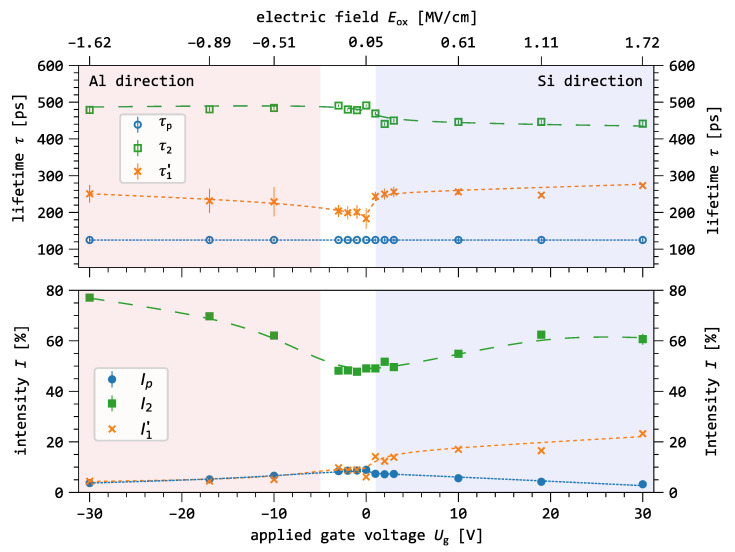
Lifetimes $\tau_{p}$ (blue), $\tau_{2}$ (green) and intensities $I_{p}$, $I_{2}$ as a function of the gate voltage $U_{g}$. The estimated oxide component, $\tau_{1}^{\prime }$ and $I_{1}^{\prime }$ from Equation (2), is shown in orange. The drift directions are divided into Al direction (red), flatband range (white), and Si direction (blue). The dashed lines are eye-guides.

As we elaborated in the previous section, the suppression of positronium formation by the drift effect leads to the generation of free positrons that can be trapped in defects. This is evidenced by the increase in the intensity $I_{2}$ of the vacancy cluster component in both drift directions (Figure 5 and Figure 6). Additionally, we identified an increase in the short component for positive gate voltages, which suggests that an unknown defect lifetime is superimposed on the p-Ps annihilation and cannot be resolved through conventional PALS analysis. Therefore, we apply the simple model from Equation (2) to extract the unresolved lifetime ($\tau_{1}^{\prime }$ and $I_{1}^{\prime }$) from the short component. Figure 7 shows the estimation of $\tau_{1}^{\prime }$ and $I_{1}^{\prime }$ over the whole gate voltage range. The calculated values are listed in Table 3.

The observations presented in Figure 7 can be classified into three distinct regions, each corresponding to a different positron drift regime:

1. Al direction ($-30\;V\leq U_{g}\leq -10\;V$): $\tau_{1}^{\prime }$ remains quasi-constant with an average lifetime of 301(7)ps and average intensity $I_{1}^{\prime }$ = 2.8(3)%. This is also the case for the medium oxide lifetime with an average value of $\tau_{2}=481\left(2\right)\;\mathrm{ps}$. The intensity $I_{2}$ decreases from 77.1(5)% to 62.0(3)%.
2. Flatband range ($-3\;V\leq U_{g}\leq 0\;V$): $\tau_{1}^{\prime }$ and $I_{1}^{\prime }$ is quasi-constant at 236(2)ps and 5(2)%, respectively. This is also observed for the medium oxide component with mean values 485(5)ps and 48(4)%.
3. Si direction ($1\;V\leq U_{g}\leq 30\;V$): $\tau_{1}^{\prime }$, remains quasi-constant with a mean value of 274(7)ps whereas the intensity, $I_{1}^{\prime }$, increases from 10.8% to 21.7%. The medium oxide lifetime, $\tau_{2}$, remains quasi-constant with a mean value of 445(5)ps, whereas the intensity, $I_{2}$, slowly increases from 49(20)% at 1V to 61(2)% for $U_{g}\geq 20\;V$.

Both lifetimes remain below the Ps average lifetime limit of 500ps at any gate voltage [30]. The variation of those two components with the applied electric field shows that free positrons can probe different electronic environments in the amorphous SiO_2_ network.

#### 3.4.4. Microstructure of the Buried Oxide

Analyzing the influence of positron drift on the lifetime spectra enables insights into the microstructure of the oxide that are not accessible under zero-field conditions, where o-Ps annihilation dominates the spectra and obscures other annihilation channels.

In Figure 7 the correlation of the lifetime components ($\tau_{1}^{\prime }$, $I_{1}^{\prime }$, $\tau_{2}$ and $I_{2}$) with the gate voltage $U_{g}$ is consistent with the existence of positron annihilation states arising from two categories of sub-nano void populations.

- Population of smaller defects $V_{s}$ related to $\tau_{1}^{\prime }$ and $I_{1}^{\prime }$.
- Population of larger defects (voids) $V_{b}$ related to $\tau_{2}$ and $I_{2}$.

The strength and direction of the electric field influence the type of voids where positrons annihilate. At higher electric fields, between 0.5–1.7 MV/cm, the size of the open spaces in the two types of sub-nanometer voids tends to be smaller when positrons drift toward silicon (Si) compared to aluminum (Al). This is shown by shorter lifetimes related to $V_{s}$ and $V_{b}$ when the gate voltage changes from $U_{g}=-30\;V$ to $U_{g}=30\;V$. For the smaller voids ($V_{s}$), the lifetime increases by 9%, accompanied by an intensity increase of up to ≈700%. In contrast, for the larger voids ($V_{b}$), the lifetime decreases by 7%, while the intensity drops by approximately 24%. This difference in void size and its effect on positron annihilation shows that the electric field gives insight into uneven distributions of the sub-nanometer voids. As the internal field strength decreases from the Al direction to the flatband range, the lifetime $\tau_{1}^{\prime }$ linked to the $V_{s}$ voids drops clearly, from 301ps to 239ps. This decrease of approximately 20% remains unclear.

As previously stated, free positrons exhibit a higher capture rate in the smaller void population ($V_{s}$) when drifted towards the SiO_2_/Si interface than when drifted towards the Al/SiO_2_ interface. This behavior can have multiple possible explanations.

One is the gradual densification along the oxidation direction, from the Al/SiO_2_ interface to the SiO_2_/Si interface [21]. Another possibility is enhanced trapping in near-interface defects, such as silicon dangling bonds ($P_{b}$ centers) [44,45]. $P_{b}$ centers at the interface of thermally oxidized, p-doped Si(100) have already been identified as possible interface defects using two-dimensional angular correlation spectroscopy (2D-ACAR) [46]. Two mechanisms may explain the increase in intensity $I_{1}^{\prime }$. First, positron drift could simply transport more positrons to the interface region, leading to increased trapping at $P_{b}$ centers. Second, electrons accumulated on the silicon side in inversion may change the charge state of $P_{b}$ centers through charge carrier trapping. Since positrons are insensitive to positively charged defects, this charge state switching could increase trapping by converting previously positive $P_{b}$ centers into effective trapping sites. However, based on current data, a definitive explanation for the increase in the intensity of the small void population cannot be given.

In summary, when the electric field is strong enough to suppress positronium formation, the open volume size and the density of voids primarily govern free positron trapping and annihilation. In cases where positronium annihilation dominates, other annihilation channels remain hidden. These channels become accessible only when the positron drift reduces the formation probability of positronium.

## 4. Conclusions

Drift-assisted Positron Annihilation Lifetime Spectroscopy (PALS) was performed on a Metal-Oxide-Silicon capacitor under gate bias of up to ±30V. The maximum achieved electric field in the 180(10)nm SiO_2_ layer was 1.72MV/cm. Positron lifetime spectra were recorded as a function of implantation energy and applied gate voltage, and analyzed using a three-component decomposition. The lifetime analysis revealed clear evidence of electric field-induced positron drift within the buried oxide of the MOS capacitor.

The most significant finding was a pronounced suppression of positronium (Ps) formation with increasing field strength, leading to enhanced annihilation of free positrons. This suppression uncovered two distinct populations of sub-nanometer voids that are otherwise obscured by Ps annihilation. As the electric field increases, the o-Ps contribution diminishes while annihilation in one or both void populations becomes more prominent. Moreover, a clear dependence on the direction of the electric field was observed. Drift towards the Al/SiO_2_ interface predominantly enhanced trapping in the larger voids, with a mean lifetime of 481(3)ps. Drift towards the SiO_2_/Si interface also increased annihilation in this population, albeit with a shorter mean lifetime of 445(3)ps. Notably, this drift direction additionally revealed a second, smaller void population, characterized by an average lifetime of 275(7)ps, which did not appear during drift towards the Al interface.

The observed directional dependencies in the lifetimes and intensities indicate spatial inhomogeneities in the distribution of sub-nanometer voids within the buried oxide layer. This shows the potential of this method for future application on oxide/semiconductor layered system using novel oxide materials, such as HfO_2_ or Gd_2_O_3_.

## Figures and Tables

**Table 1:** Summary of the applied gate voltage steps and their corresponding electric field over the 180(10)nm oxide layer.

$U_{g}\left[V\right]$	−30	−17	−10	−3	−2	−1	0	1	2	3	10	19	30
$E_{\mathrm{ox}}\left[\mathrm{MV}/\mathrm{cm}\right]$	−1.62	−0.89	−0.51	−0.12	−0.06	−0.01	0.05	0.11	0.16	0.22	0.61	1.11	1.72

**Table 2:** Summary of the results of the positron lifetime analysis for the three-component decomposition for the buried (MOS) and the bare oxide. The same initial fit parameters were used for all lifetime spectra. The uncertainty of the gate voltage is ±0.5V.

$U_{g}$	$E_{\mathrm{ox}}$	$\tau_{1}$	$I_{1}$	$\tau_{2}$	$I_{2}$	$\tau_{3}$	$I_{3}$
[V]	[MV/cm]	[ps]	[%]	[ps]	[%]	[ps]	[%]
−30	−1.62	193(8)	8.1(6)	479(2)	77(1)	1443(5)	15(1)
−17	−0.89	175(5)	9.7(4)	481(2)	70(1)	1463(3)	21(1)
−10	−0.51	170(4)	12(1)	484(2)	62(1)	1477(3)	26(1)
−3	−0.12	167(3)	18(1)	491(3)	48(1)	1488(3)	34(2)
−2	−0.06	162(3)	17(1)	480(3)	48(1)	1480(3)	34(2)
−1	0.01	164(3)	18(1)	478(3)	48(1)	1480(3)	35(2)
0	0.05	149(3)	15(1)	491(3)	49(1)	1488(3)	36(2)
1	0.11	203(4)	22(1)	469(4)	49(1)	1490(3)	29(2)
2	0.16	204(5)	19(1)	441(4)	52(1)	1480(3)	29(1)
3	0.22	211(5)	21(1)	449(5)	50(1)	1484(3)	29(2)
10	0.61	224(5)	23(1)	446(4)	55(1)	1479(3)	23(1)
19	1.11	222(5)	21(1)	447(4)	62(1)	1465(4)	17(1)
30	1.72	255(7)	26(2)	442(5)	61(2)	1456(5)	13(1)
bare oxide	-	137(1)	23(1)	595(6)	25(1)	1563(3)	52(1)

**Table 3:** Evolution of the short oxide component, $\tau_{1}^{\prime }$ and $I_{1}^{\prime }$ calculated with Equation (2), and the mean values of the second component, ${\bar{\tau }}_{2}$ and ${\bar{I}}_{2}$, as a function of gate voltage. The relevant drift ranges for the evolution of the two components are color-coded: Al direction (blue), flatband range (white), and Si direction (red).

$U_{g}$	$E_{\mathrm{ox}}$	$\tau_{1}^{\prime }$	$I_{1}^{\prime }$	${\bar{\tau }}_{2}$	${\bar{I}}_{2}$
[V]	[MV/cm]	[ps]	[%]	[ps]	[%]
−30	−1.62	299(24)	3.2(6)	481(3)	69(7)
−17	−0.89	297(33)	2.8(4)	481(3)	69(7)
−10	−0.51	308(40)	2.8(4)	481(3)	69(7)
−3	−0.12	236(16)	7.0(4)	485(6)	48(4)
−2	−0.06	235(18)	5.8(4)	485(6)	48(4)
−1	0.01	237(18)	6.1(4)	485(6)	48(4)
0	0.05	238(27)	3.2(3)	485(6)	48(4)
1	0.11	268(12)	12(1)	445(11)	55(3)
2	0.16	280(15)	10(1)	445(11)	55(3)
3	0.22	285(14)	12(1)	445(11)	55(3)
10	0.61	271(8)	15(1)	445(11)	55(3)
19	1.11	258(6)	15(1)	445(11)	55(3)
30	1.72	281(4)	22(1)	445(11)	55(3)

## Data Availability

The data presented in this study are available on request from the corresponding author due to planned follow-up research.
